# Sequencing of neurofilament genes identified *NEFH* Ser787Arg as a novel risk variant of sporadic amyotrophic lateral sclerosis in Chinese subjects

**DOI:** 10.1186/s12920-021-01073-z

**Published:** 2021-09-11

**Authors:** Feng Lin, Wanhui Lin, Chaofeng Zhu, Jilan Lin, Junge Zhu, Xu-Ying Li, Zhanjun Wang, Chaodong Wang, Huapin Huang

**Affiliations:** 1 Department of Neurology, Fujian Medical University Union Hospital, Fujian, 350001 China; 2 Department of Neurology, Xuanwu Hospital of Capital Medical University, Beijing, 100053 China

**Keywords:** Sporadic amyotrophic lateral sclerosis, Neurofilament genes, Rare variant, Association

## Abstract

**Background:**

Amyotrophic lateral sclerosis (ALS) is a devastating neurodegenerative disease with neuronal cell inclusions composed of neurofilaments and other abnormal aggregative proteins as pathological hallmarks. Approximately 90% of patients have sporadic cases (sALS), and at least 4 genes, i.e. *C9orf72*, *SOD1*, *FUS* and *TARDBP*, have been identified as the main causative genes, while many others have been proposed as potential risk genes. However, these mutations could explain only ~ 10% of sALS cases. The neurofilament polypeptides encoded by *NEFH*, *NEFM,* and *NEFL* are promising protein biomarkers for ALS and other degenerative diseases. However, whether the genetic variants of these genes were associated with ALS remain ambiguous.

**Methods:**

Here, we used PCR-Sanger to sequence the exons of these three genes in a cohort of 371 sALS patients and 711 healthy controls (Phase I) and validated the risk variant in another 300 sALS patients and 1076 controls (Phase II).

**Results:**

A total of 92 variants were identified, including 36 rare heterozygous variants in *NEFH*, 27 in *NEFM,* and 16 in *NEFL*, and only rs568759161 (p.Ser787Arg) in *NEFH* reached nominal statistical power (*P* = 0.02 at Phase I, *P* = 0.009 at Phase II) in the case–control comparison. Together, the Phase I and II studies showed the significantly higher frequency of the variant in cases (9/1342, 0.67%) than in controls (2/3574, 0.07%) (OR 12.06; 95% CI 2.60–55.88; *P* = 0.0003). No variants passed multiple testing in the discovery cohort, but rs568759161 was associated with ALS in a replication cohort.

**Conclusions:**

Our results confirmed that *NEFH* Ser787Arg is a novel sALS risk variant in Chinese subjects, but *NEFM* and *NEFL* were not associated with sALS. These data may have implications for genetic counselling and for understanding the pathogenesis of sALS.

**Supplementary Information:**

The online version contains supplementary material available at 10.1186/s12920-021-01073-z.

## Introduction

Amyotrophic lateral sclerosis (ALS) is a devastating neurodegenerative disease characterized by loss of motor neurons in the brain and spinal cord, resulting in muscle atrophy, swallowing disorders, and pyramidal tract signs. A known pathological hallmark of ALS is neuronal cell inclusions composed of neurofilaments and other abnormal aggregative proteins [1–3]. Epidemiological surveys show an incidence of 0.6–3.8 per 100,000 persons per year and a prevalence of 4.1–8.4 per 100,000 persons worldwide [4]. It has been reported that the yearly incidence is 0.8 (2010–2015) per 100,000 persons in China [5]. Approximately 10% of cases were familial, and 90% were sporadic cases. To date, the genetics of ALS are not fully understood. In 1993, *SOD1* was discovered as the first ALS-causing gene. Since then, many other genes have been reported to be causative for (i.e. *C9orf72*, *SOD1*, *FUS*, *TARDBP*, etc.) or associated with the disease [6, 7]. Genetic studies have found that mutations in these genes were mainly identified in familial cases and could explain only approximately 10% of sporadic cases (sALS) [6]. With next-generation sequencing, novel genes and loci have been increasingly discovered [8], but the genetics of sALS are not fully understood.

Neurofilaments (NFs) are type IV intermediate filament heteropolymers composed of light (NEFL), medium (NEFM), and heavy (NEFH) subunits. The different NF subunits have the same conserved alpha-helical rod domain and differ in the head and tail domains. NFs function by determining axonal calibre, promoting axonal growth, and forming a 3-dimensional lattice that supports cytoplasmic organelle organization [9]. NFs have been considered to play an essential role in many neurodegenerative diseases, such as ALS, Charcot-Marie-Tooth disease, and Parkinson's disease [10]. Many studies have revealed the relationship between NFs and ALS. First, one of the important pathological features of ALS is the cytoplasmic inclusion bodies of NFs [11–14]. Second, motor function impairment is observed in NF-subunit-transgenic mice [15, 16]. Third, studies have found a decrease in *NEFL* mRNA expression in the spinal cord tissue of patients with ALS [17, 18]. Recently, NEFL and phosphorylated NEFH (pNEFH) were considered as promising novel biomarkers in the blood and cerebrospinal fluid of ALS patients during disease onset and progression [19–21]. In previous studies, *NEFH* variants were reported in approximately 1% sALS cases [22–26], and *NEFH* was considered a susceptibility gene for ALS. However, the conclusions from many different studies are contradictory [27–29]. The mechanism may involve abnormal protein modification, folding, clearance, and axonal transport [30]. However, *NEFM* and *NEFL* have not been linked to ALS, although upregulation of NEFM has been detected in spinal cord tissues in patients with ALS or ALS-like diseases [31, 32]. Thus far, systemic sequencing studies of *NEFH*/*NEFM*/*NEFL* with large samples have been rare, and most of the relevant studies have been restricted to the Caucasian population. Moreover, the distribution, burden, and significance of these genetic variations remain ambiguous, especially in the Chinese sALS cohort.

In this study, we sequenced the variants in the exons of *NEFH*, *NEFM,* and *NEFL* in a Chinese sALS cohort including 371 sALS patients and 711 healthy controls (Phase I) to identify the potentially associated variants and validate these variants in another 300 sALS patients and 1076 controls (Phase II). We found that rs568759161 (p.Ser787Arg) in *NEFH* was a novel risk variant associated with sALS, and the distribution of this genetic variant was different from that observed in previous studies. However, *NEFM* and *NEFL* were not definitively associated with sALS.

## Materials and methods

### Patients and controls

A total of 371 sALS patients and 711 healthy subjects of Han ethnicity were recruited from the Department of Neurology of three hospitals (Fujian Medical University Union Hospital, Sanming First Hospital Affiliated to Fujian Medical University, and Xuanwu Hospital of Capital Medical University) from Jan 2016 to Nov 2020 (Phase I). Another 1076 healthy elderly control subjects were recruited from communities in Beijing as a further validation control group, and 300 sALS patients were recruited from the aforementioned hospitals to confirm the risk variant (Phase II). The inclusion criteria of the control group were healthy elderly people without diseases history of motor neuron diseases, degenerative neurological disorders or malignancy.

All patients with ALS were diagnosed by at least two neuromuscular specialists in each hospital based on clinical and electrophysiological findings according to the revised El Escorial criteria [33]. We only recruited sporadic cases in the study, which were defined as the absence of a second patient within three generations of the family, and frontotemporal dementia (FTD) was excluded in each patient in this study. And all sALS patients fulfilled the criteria for probable, or definite ALS based on this criterion. Clinical data, including age at onset (AAO), initial site of impairment, core symptoms and signs, electromyography, and nerve conduction velocity assessment, were reviewed and analysed reciprocally by researchers and specialists from the hospitals. Family inquiry was performed to exclude the existence of kinship among samples within at least three generations.

### NEFH/NEFM/NEFL genetic analysis

Three millilitres of blood were obtained from sALS patients and controls. Polymerase chain reaction (PCR) assays and extension primers for exons were designed using Oligo 6.0 software (Molecular Biology Insights, Inc., CO, USA). The primer sequences for amplifying the exons of *NEFL*, *NEFM,* and *NEFH* are listed in Additional file 1: Table S1. PCR products were purified and sequenced by an ABI 3730 DNA Analyzer (Applied Biosciences, Inc., CT, USA). Chromas 2.22 software was used for sequence reading. The variant position at the genomic level was based on GenBank accession number NC_000022.10/NC_000008.10/NC_000008.10, the transcript position was based on NM_021076.3/NM_005382.2/NM_006158.4, and the protein-level position was based on NP_066554.2/NP_005373.2/NP_006149.2, according to the hg19/GRCh37 reference sequence. The chromosomal position, frequencies, and other relevant information of the variants were annotated using the 1000 Genome Project database (http://www.1000genomes.org), dbSNP version 147 (http://www.ncbi.nlm.nih.gov/projects/SNP), and the Exome Aggregation Consortium (ExAC) (http://exac.broadinstitute.org/) database. Variants were classified into the following categories according to their minor allele frequencies (MAFs): MAF > 0.05, common variant; 0.01 ≤ MAF ≤ 0.05, low-frequency variant; and MAF < 0.01, rare variant. We paid close attention to the rare variant (MAF < 0.01). The ExAC_EAS (for the East Asian population) and GnomAD_exome_EAS databases were used as references. Novel variants were defined as those that were not indexed in any of the databases, irrespective of ethnic population. The functional effect of the variants was predicted by combined annotation-dependent depletion (CADD) (https://cadd.gs.washington.edu/snv).

### Statistical analysis

Low-frequency and common variants located in the *NEFH*, *NEFM,* and *NEFL* coding regions in the control group were tested for deviations from Hardy–Weinberg equilibrium using the χ^2^ test. Allele frequencies of common and low-frequency variants in patients and controls were compared by χ^2^ statistics using SPSS 22 software. Nominal *P* values were corrected for the number of variants tested using Bonferroni correction. The burden test for rare coding variants across the full *NEFH*, *NEFM,* and *NEFL* coding sequences was performed by the sequence kernel association test (SKAT-O) using R software (version 4.0.0). Differences in AAO between patients carrying and not carrying rare variants were calculated using an unpaired nonparametric (Mann–Whitney) test. *P* < 0.05 was considered statistically significant.

## Results

### Demographic data of the cohort

The cohort consisted of two phases of cases and controls. Phase I included 371 patients with sALS and 711 healthy controls. The AAO of the patients was 53.42 ± 10.28 years. Phase II included another 300 sALS patients and 1076 controls. The AAO was 53.49 ± 9.56 years in the cases. It has been reported that ALS was more prevalent in men, and the mean AAO was 51 (IQR 43–59) years in China [34]. Therefore, we chose more female individuals in the controls, who were older (69.41 ± 8.42 and 69.83 ± 7.70 years) than the cases selected (Table 1).

**Table 1:** Demographic data of the study subjects

Clinical features	Phase I		Phase II		Combined	
	sALS (n = 371)	Control (n = 711)	sALS (n = 300)	Control (n = 1076)	sALS (n = 671)	Control_2 (n = 1787)
Sex, M/F (ratio)	227/144 (1.58:1)	283/428 (0.66:1)	183/117 (1.56:1)	453/623 (0.73:1)	410/261 (1.57:1)	736/1051 (0.70:1)
Age (year, mean ± SD)	55.13 ± 10.28	69.41 ± 8.42	55.16 ± 9.82	69.83 ± 7.70	55.14 ± 9.97	69.66 ± 7.91
Age at onset (year, mean ± SD)	53.42 ± 10.28	–	53.49 ± 9.56	–	53.45 ± 9.96	–
Site of onset, bulbar (%)	73/371 (19.68%)	–	59/300 (19.61)	–	132/671 (19.67%)	–

sALS, sporadic amyotrophic lateral sclerosis; AAO, age at onset

### Rare coding variants identified in the NEFH, NEFM, and NEFL genes

**Fig. 1:**
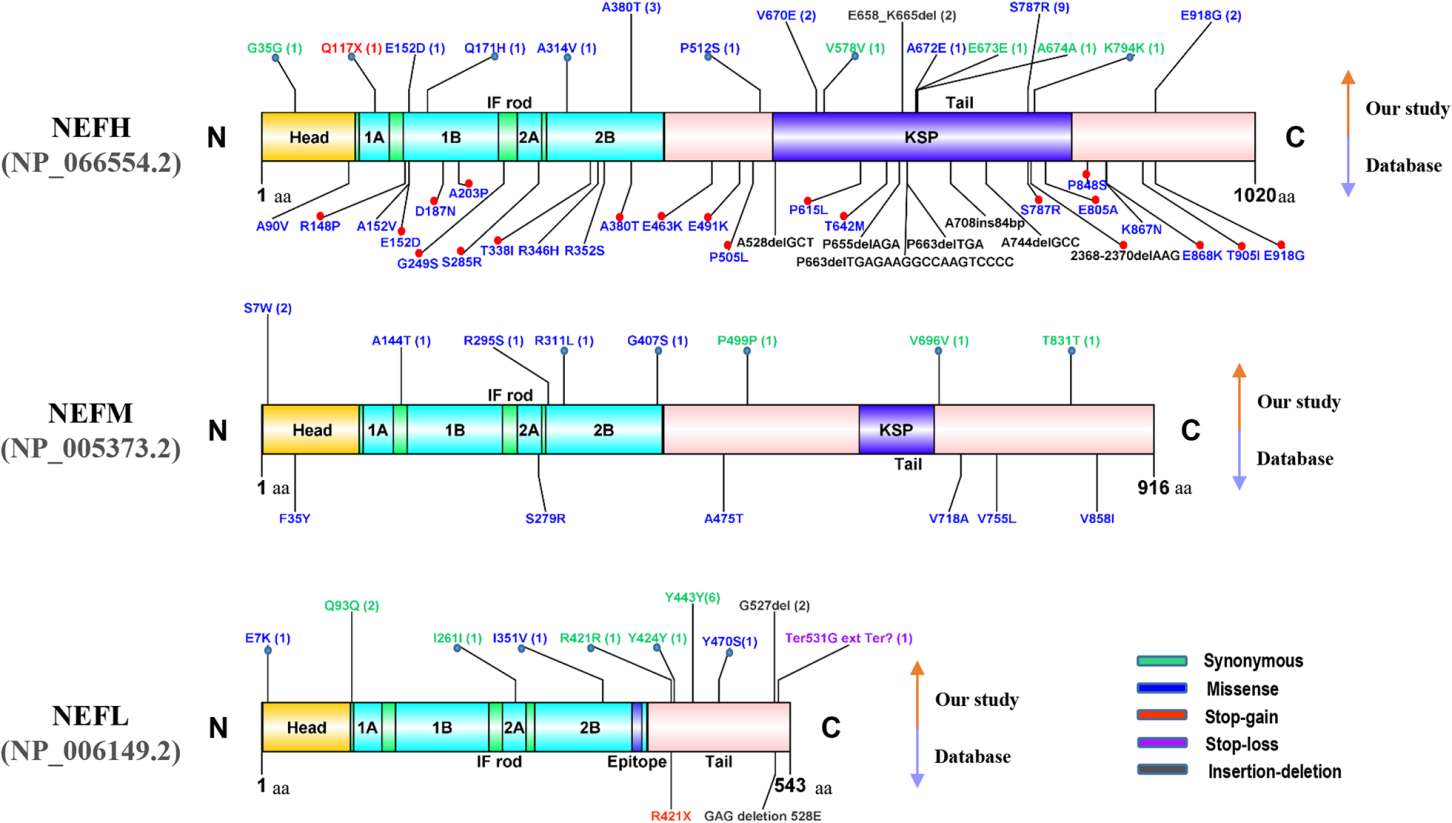
Comparison of the variants found in our research and previous literature reports. Numbers at the end of the variant name represent the number of patients. Variants detected in cases only are indicated by blue dots. Variants detected in next generation of sequencing (NGS) are indicated by red dots. 1A, 1B, 2A, 2B: The Coil 1A, Coil 1B, Coil 2A, Coil 2B regions, respectively; Rod: intermediate filament rod; KSP repeats, repeats of lysine-serine-proline; Epitope: recognized by an IF-specific monoclonal antibody; SubA, SubB: subdomain A, subdomain B (acidic). The amino acid position and functional domains are depicted according to the UniProt database (http://www.uniprot.org/uniprot/). The plot was created with DOG v. 2.0 software (http://dog.biocuckoo.org/). Abbreviations: ALS, amyotrophic lateral sclerosis; NEFH, neurofilament heavy polypeptide. NEFM, neurofilament medium polypeptide. NEFL neurofilament light polypeptide

We screened the exons and their flanking sequences in the *NEFH*, *NEFM, and NEFL* genes by PCR and Sanger sequencing (Additional file 6: Fig. S1). We identified 92 variants, including 36 rare heterozygous variants in *NEFH*, 27 in *NEFM*, and 16 in *NEFL*. There were 16 rare coding variants of *NEFH* in 20 sALS cases, 8 of *NEFM* in 9 sALS cases, and 10 of *NEFL* in 17 sALS cases. The rare variants included 1 stop-gain, 1 stop-loss, 4 frameshift, 2 insertion/deletion, 44 missense, 1 intron-harboured, and 26 synonymous variants in 44 sALS cases (20 cases carried rare variants in NEFH, 9 in NEFM, and 17 in NEFL). Of the sALS patients who carried rare variants, 2 had variants in two genes. Of the rare nonsynonymous variants, 4 in *NEFH*, 2 in *NEFM,* and 3 in *NEFL* were identified only in cases, while 14 in *NEFH*, 13 in *NEFM,* and 5 in *NEFL* were identified only in control subjects. Seven variants in *NEFH*, 3 in *NEFM,* and 2 in *NEFL* were identified in both the cases and controls. Given these genes, 9 heterozygous missense mutations, 1 in-frame deletion, and 1 nonsense mutation in *NEFH* were distributed in 4 exons, especially in exons 4 and 1; 5 missense variants were distributed in exons 3 and 1 in *NEFM*; and 1 stop-loss, 1 in-frame deletion and 3 missense variants were distributed in the exons of *NEFL* (Fig. 1). Upon comparing the cases and controls, we found that only one variant, rs568759161 (c.2361C > G, p.Ser787Arg), in *NEFH* was nominally more frequent in cases than in controls (OR 9.64; 95% CI 1.12–82.67; *P* = 0.02) (Table 2 and Additional file 2: Table S2). However, no variants passed the Bonferroni multiple comparison test.

**Table 2:** Rare nonsynonymous coding variants identified in *NEFH*

Group	Chr_Position	dbSNP	cDNA_change	AA _change	ExAC_EAS	gnomAD_exome_EAS	CADD	No. Carriers (n = 371)	No. Controls (n = 711)	*P* value	OR (95% CI)
Case only	22:29,876,600	–	c.349C > T	p.Gln117Ter	–	7.54E-05	Damaging	1	0	0.34	Inf (Na-Inf)
	22:29,876,764	–	c.513G > C	p.Gln171His	–	–	Tolerable	1	0	0.34	Inf (Na-Inf)
	22:29,879,421	rs539511579	c.941C > T	p.Ala314Val	2.32E-04	1.74E-04	Damaging	1	0	0.34	Inf (Na-Inf)
	22:29,885,163	–	c.1534C > T	p.Pro512Ser	–	–	Damaging	1	0	0.34	Inf (Na-Inf)
Control only	22:29,876,270	–	c.19G > A	p.Ala7Thr	–	–	Damaging	0	1	1.00	Na
	22:29,876,409	rs772280985	c.158C > T	p.Thr53Met	0.00	0.00	Damaging	0	1	1.00	Na
	22:29,876,520	rs61556467	c.269C > T	p.Ala90Val	0.00	0.00	Damaging	0	1	1.00	Na
	22:29,876,710	rs763364083	c.469_491del	p.Val157ArgfsTer115	0.00	1.29E-04	–	0	1	1.00	Na
	22:29,879,444	rs778265423	c.964C > T	p.Arg322Trp	0.00	0.00	Damaging	0	1	1.00	Na
	22:29,884,842	rs200464796	c.1213C > A	p.Leu405Ile	0.00	0.00	Damaging	0	1	1.00	Na
	22:29,885,182	–	c.1553C > T	p.Ser518Leu	–	–	Damaging	0	1	1.00	Na
	22:29,885,198	rs138278265	c.1569G > C	p.Glu523Asp	0.00	0.00	Tolerable	0	1	1.00	Na
	22:29,885,215	–	c.1586A > C	p.Glu529Ala)	–	–	Tolerable	0	1	1.00	Na
	22:29,885,735	–	c.2106_2110del	p.Lys703ProfsTer2	–	–	–	0	1	1.00	Na
	22:29,885,741	–	c.2112_2232del	p.Pro705SerfsTer17	–	–	–	0	1	1.00	Na
	22:29,885,917	–	c.2288C > T	p.Ser763Phe	–	–	Damaging	0	1	1.00	Na
	22:29,886,118	rs201757428	c.2489C > T	p.Pro830Leu	4.81E-04	2.91E-04	Tolerable	0	2	0.54	Na
	22:29,886,426	rs777317391	c.2797C > T	p.Pro933Ser	9.53E-04	1.33E-03	Tolerable	0	1	1.00	Na
Both	22:29,876,707	rs774792100	c.456G > C	p.Glu152Asp	7.46E-03	4.57E-03	Tolerable	1	1	1.00	1.92 (0.12–30.70)
	22:29,881,766	rs201416955	c.1138G > A	p.Ala380Thr	5.78E-03	5.33E-03	Damaging	3	8	0.76	0.72 (0.19–2.71)
	22:29,885,581	rs267607533	c.1965_1988del	p.Glu658_Lys665del	1.16E-04	8.37E-03	–	2	6	0.72	0.64 (0.13–3.17)
	22:29,885,638	rs190692435	c.2009 T > A	p.Val670Glu	1.17E-04	1.91E-03	Tolerable	2	5	1.00	0.77 (0.15–3.96)
	22:29,885,644	–	c.2015C > A	p.Ala672Glu	1.17E-04	1.60E-03	Tolerable	1	5	0.67	0.38 (0.05–3.28)
	22:29,885,990	rs568759161	c.2361C > G	p.Ser787Arg	1.39E-03	2.38E-03	Damaging	5	1	0.02	9.64 (1.12–82.67)
	22:29,886,382	rs189881592	c.2753A > G	p.Glu918Gly	3.34E-03	3.53E-03	Damaging	2	2	0.61	1.92 (0.27–13.65)

cDNA-level nomenclature was based on NM_021076.3. According to hg19/GRCh37, protein-level nomenclature was based on NP_066554.2; dbSNP, accession number of the variant in the Database of Single-Nucleotide Polymorphisms 147; MAF, minor allele frequency; ExAC_EAS and GnomAD_exome_EAS, MAFs of variants in the Exome Aggregation Consortium (ExAC) and GnomAD_exome databases for the East Asian population; In silico prediction by Combined Annotation Dependent Depletion (CADD); AA, amino acid; Ifn, infinity; 95% CI, 95% confidence interval; Na, not available,. A value of *P* < 0.05 was considered statistically significant (*P* < 0.0005 after Bonferroni correction)

### Burden test for rare variants of NEFH and NEFL in ALS

To investigate the enrichment of rare coding variants in ALS, we performed the SKAT-O burden test for each gene. We chose the dominant inheritance (Dom) model for nonsynonymous coding variants and the non-benign and loss-of-function (LoF) variants. As shown in Additional file 3: Table S3, none of the genes showed significant enrichment of rare variants in cases.

### Validation of the association of NEFH Ser787Arg variant with sALS

No variants passed multiple testing in the discovery cohort, in which the only rare variant, rs568759161 (p.Ser787Arg), in NEFH, was nominally associated with ALS. This variant was found in 5 sALS patients and 1 control in Phase I (MAF: 5/742, 0.67% and 1/1422, 0.07%; *P* = 0.02; OR 9.64; 95% CI 1.12–82.67). Due to the lack of significance in the Bonferroni correction, we added 300 cases and 1076 controls for sequencing (Phase II validation). The variant was found in 4 sALS patients and 1 control in Phase II (MAF: 4/600, 0.67% and 1/2152, 0.05%; *P* = 0.009; OR 14.43; 95% CI 1.61–129.40). Upon combining the two phases, the variant was shown to be significantly more abundant in cases than in controls (OR 12.06; 95% CI 2.60–55.88; *P* = 0.0003) (Table 3).

**Table 3:** Statistical outcome in variant (NEFH p.Ser787Arg) carriers

Variables	Phase I		Phase II		Combined	
	sALS (n = 371)	Control (n = 711)	sALS (n = 300)	Control (n = 1076)	sALS (n = 671)	Control_2 (n = 1787)
No. carriers	5	1	4	1	9	2
MAF	6.73E-03	7.03 E-04	6.67E-03	4.65 E-04	6.71E-03	5.60 E-04
*P* value	0.02		0.009		0.0003	
OR (95% CI)	9.64 (1.12–82.67)		14.43 (1.61–129.40)		12.06 (2.60–55.88)	

sALS, sporadic amyotrophic lateral sclerosis; MAF, minor allele frequency; *P* value, determined using Fisher's exact test; 95% CI, 95% confidence interval

In clinical aspects, one female and 8 male sALS patients shared the NEFH p.Ser787Arg variant with AAO at 53.44 ± 13.51 years, which was not different from other sALS cases. Most (7/9) of these patients initially presented with limb symptoms (Additional file 4: Table S4).

### Low-frequency and common coding variants identified in the NEFH, NEFM, and NEFL genes

Among all the nonsynonymous variants, 5 low-frequency (0.01 ≤ MAF ≤ 0.05) were identified in NEFH, 1 was in NEFM and 1 was in NEFL. For common (MAF > 0.05) variants, 4 were revealed in NEFH, 1 was in NEFM and 1 was in NEFL (Additional file 5: Table S5). The results suggested that genotype frequencies of *NEFH*, *NEFM* and *NEFL* were Hardy–Weinberg equilibrium in the control group. However, there were no statistically significant differences in common or low-frequency variants between the case and control groups.

## Discussion

In our study, it was found that many domains harboured rare variants in *NEFH*, *NEFM,* and *NEFL* in sALS patients, which is different from the results of previous studies [35] and the ALSod database (https://alsod.ac.uk/output/gene.php#variants). Previous ALS studies have indicated that the *NEFH* mutations are insertions and deletions and are mostly located at the tail [22, 26]. Our study demonstrated more point variants than insertion/deletion variants in *NEFH* (Fig. 1). Second, our data showed more carriers with rare nonsynonymous variants (17/371, 4.58%) in *NEFH* than previous reports showed in other populations [25, 29, 36–38]. Third, mutation of *NEFL* has traditionally been recognized as a cause of Charcot-Marie-Tooth disease [39], congenital myopathy in humans [40], and motor neuron disease in mice [32]. *NEFM* is linked to Parkinson's disease [41]. However, *NEFL* and *NEFM* were not associated with sALS in our study. Although we had shown variants in these genes in sALS, we did not find significant differences in clinical characteristics (sex, AAO, onset site) between cases carrying and not carrying the variants. The differences further confirmed the genetic heterogeneity in sALS among different ethnicities and highlight the association of *NEFH*, but not *NEFL* or *NEFM*, with ALS.

This study found that the p.Ser787Arg variant in *NEFH* was associated with sALS in Chinese subjects. Notably, rs568759161 is only found in only the East Asian population according to ExAC (MAF 0.14%) and gnomAD (MAF 0.24%), and their MAFs were slightly higher than those of our control group (0.07% in Phase I and 0.05% in Phase II) (Table 3). We assume that the difference in allele frequency between the two databases and our study might be due to the population differences. Moreover, we found that some variants reported by other studies were not associated with ALS. For example, A380T in *NEFH* was identified only in cases previously [29], but our study suggested it was identified in both case and control groups. So, we believed study of rare variants need large samples of controls. In our study, we recruited relatively large controls (n = 711 in Phase I and n = 1076 in Phase II) to decrease the chance of false positive or false negative.

The phosphorylation of NF subunits has been considered a critical process regulating the formation and function of NFs [10]. The variant p.Ser787Arg is located in the phosphorylated region in a conserved sequence. Proper phosphorylation/dephosphorylation of NEFH may be considered a protective mechanism under conditions of cellular stress [16, 42], indicating that the modification of NEFH plays a significant role in maintaining the normal function of neurons. We hypothesized that the NEFH-S787R variant changes the phosphorylation of the protein. However, because of the unavailability of an antibody against the site, we did not test the hypothesis in this study. In the future, we need to synthesize antibodies against the phosphorylated NEFH-Ser787 site to further explore the changes in phosphorylation levels.

Recently, next-generation sequencing technology have identified many genes, including *NEFH* [43], as causative for or associated with ALS. In *NEFH*, 20 variants have been reported in ALS cases (Fig. 1) [25, 36–38, 44, 45], but none was conclusively related to the disease. The p.Ser787Arg was only reported by Chen et al. [45], but its association with ALS was not confirmed. Our study provided the spectrum of *NEFH* variants and confirmed the association of p. Ser787Arg with Chinese sALS.

## Conclusion

In this study, we analysed the mutational spectrum of *NEFH*, *NEFM*, and *NEFL* genes in an sALS Chinese cohort and identified the variant (rs568759161) locating in the phosphorylated site of the KSP domain of *NEFH* as a risk variant associated with sALS in Chinese. Functional studies will be necessary to assess its role in ALS pathogenesis.

## Supplementary Information

**Additional file 1**. Primers for amplification of exons.

**Additional file 2**. Rare non-synonymous coding variants in NEFM and NEFL.

**Additional file 3**. Burden in all rare variants.

**Additional file 4**. Clinical features of NEFH (S787R) carriers.

**Additional file 5**. Low frequency and common variants identified in NEFH, NEFM and NEFL genes.

**Additional file 6**. Workflow of the study design.

## Data Availability

All genetic polymorphisms identified in this study is available at Table 2, Additional file 2: Table S2 and Additional file 5: Table S5. The primer sequences are shown in Additional file 1: Table S1. The original sequencing and clinical datasets generated during the current study are not publicly available due to maintaining patient confidentiality but are available from the corresponding author (hh-p@163.com) on reasonable request.

## Abbreviations

ALS: Amyotrophic lateral sclerosis
NFs: Neurofilaments
FTD: Frontotemporal dementia
AAO: Age at onset
PCR: Polymerase chain reaction
ExAC: Exome aggregation consortium
MAFs: Minor allele frequencies
CADD: Combined annotation-dependent depletion
SKAT-O: Sequence kernel association test
LoF: Loss-of-function
