# Supplementary material for: Sequencing of neurofilament genes identified NEFH Ser787Arg as a novel risk variant of sporadic amyotrophic lateral sclerosis in Chinese subjects
Source: BMC Med Genomics. 2021 Sep 11;14:222. doi: 10.1186/s12920-021-01073-z (PMC8436554; doi:10.1186/s12920-021-01073-z)
Supplement: Supplementary file 6 — Additional file 6. Workflow of the study design. [file 12920_2021_1073_MOESM6_ESM.docx]

**Supplemental Fig. 1.** **Workflow of the study design.**


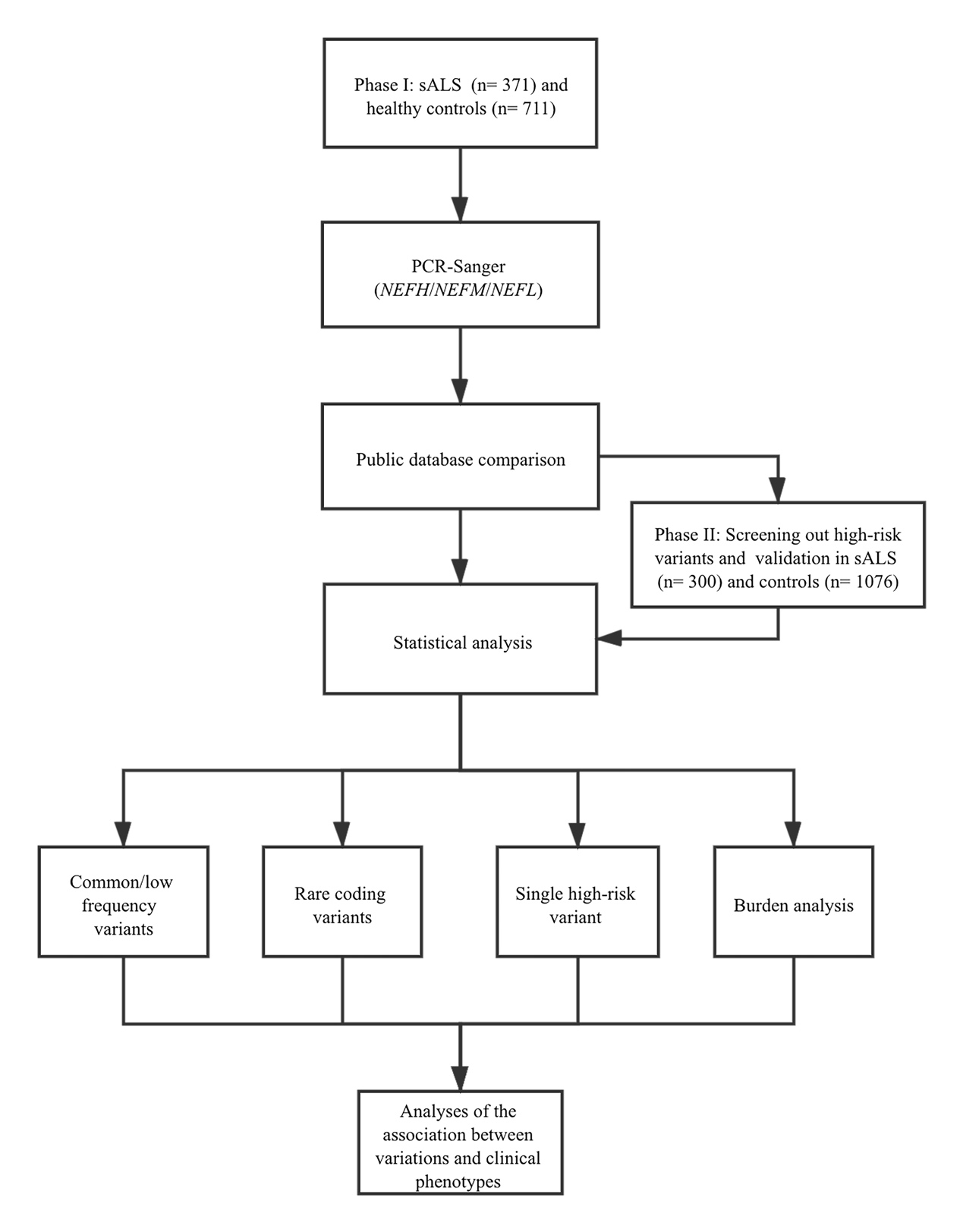


**Supplemental Fig. 1. Workflow of the study design.**

sALS, sporadic amyotrophic lateral sclerosis.
